# Listening In on the Past: What Can Otolith δ^18^O Values Really Tell Us about the Environmental History of Fishes?

**DOI:** 10.1371/journal.pone.0108539

**Published:** 2014-10-03

**Authors:** Audrey M. Darnaude, Anna Sturrock, Clive N. Trueman, David Mouillot, EIMF, Steven E. Campana, Ewan Hunter

**Affiliations:** 1 UMR CNRS-UM2-UM1-IFREMER-IRD 5119 Ecologie des Systèmes Marins Côtiers, Montpellier 2 University, Montpellier, France; 2 Centre for Environment, Fisheries and Aquaculture Science, Lowestoft Laboratory, Lowestoft, United Kingdom; 3 Ocean and Earth Science, National Oceanography Centre, University of Southampton, Southampton, United Kingdom; 4 Edinburgh Ion Microprobe Facility, School of GeoSciences, University of Edinburgh, Edinburgh, United Kingdom; 5 Population Ecology Division, Bedford Institute of Oceanography, Dartmouth, Canada; Department of Agriculture, Australia

## Abstract

Oxygen isotope ratios from fish otoliths are used to discriminate marine stocks and reconstruct past climate, assuming that variations in otolith δ^18^O values closely reflect differences in temperature history of fish when accounting for salinity induced variability in water δ^18^O. To investigate this, we exploited the environmental and migratory data gathered from a decade using archival tags to study the behaviour of adult plaice (*Pleuronectes platessa* L.) in the North Sea. Based on the tag-derived monthly distributions of the fish and corresponding temperature and salinity estimates modelled across three consecutive years, we first predicted annual otolith δ^18^O values for three geographically discrete offshore sub-stocks, using three alternative plausible scenarios for otolith growth. Comparison of predicted *vs*. measured annual δ^18^O values demonstrated >96% correct prediction of sub-stock membership, irrespective of the otolith growth scenario. Pronounced inter-stock differences in δ^18^O values, notably in summer, provide a robust marker for reconstructing broad-scale plaice distribution in the North Sea. However, although largely congruent, measured and predicted annual δ^18^O values of did not fully match. Small, but consistent, offsets were also observed between individual high-resolution otolith δ^18^O values measured during tag recording time and corresponding δ^18^O predictions using concomitant tag-recorded temperatures and location-specific salinity estimates. The nature of the shifts differed among sub-stocks, suggesting specific vital effects linked to variation in physiological response to temperature. Therefore, although otolith δ^18^O in free-ranging fish largely reflects environmental temperature and salinity, we counsel prudence when interpreting otolith δ^18^O data for stock discrimination or temperature reconstruction until the mechanisms underpinning otolith δ^18^O signature acquisition, and associated variation, are clarified.

## Introduction

Ecological studies in offshore marine ecosystems are often complicated by a lack of information describing the ambient environmental conditions habitually experienced by resident populations. Natural proxies that indirectly record the environmental conditions experienced by marine organisms, such as the isotopic ratio of oxygen in biogenic carbonates (expressed as δ^18^O values) are therefore particularly valuable for providing long-term ecological insights into marine environments [1]. The isotopic composition of oxygen in biogenic carbonates is influenced by both temperature and the isotopic composition of the ambient water [2]. However, because water δ^18^O signature is primarily salinity dependent, it is assumed to remain effectively constant in offshore water masses [3], where the δ^18^O values of organisms' calcified structures is gaining increasing recognition as a proxy for temperature [1]. Since the pioneering study of [4], the isotopic composition of oxygen in fish otoliths (“ear-stones”) has thus been commonly applied as a proxy for seawater temperature both by ecologists (e.g. [5]–[7]) and paleontologists (e.g. [8]–[11]).

Otoliths are calcified structures located within the inner ears of teleost fish [12] which grow continuously from birth, forming seasonal accretion increments whose chemical composition reflects ambient water characteristics at the time of deposition, at least for some elements and isotopes [13]. Because otolith material is not resorbed or physiologically altered [12], otoliths offer natural data storage, providing a retrospective, temporally resolved record of lifetime environmental history through their structure and chemistry, often more detailed than the other calcified structures commonly used in aquatic ecology or paleontology [8], [14]. The universal presence of teleost fish in aquatic ecosystems, and the ubiquity of otoliths in the fossil record from the late Cretaceous to present [15], gives otoliths enormous potential value in interpreting past environmental conditions and understanding current climate change [1], mainly through the generation of individual-specific lifelong records of temperature history (e.g. [6], [16], [17]). Given the wide range of ocean temperatures [18], otolith δ^18^O values can also provide a relatively low-cost tool for substantially improving our knowledge of fish spatial dynamics and population structure for the effective conservation and sustainable exploitation of marine fish stocks [19]. To date, otolith δ^18^O values have allowed successful identification of marine fish origin (e.g. [20], [21]) differentiation between resident and migrant fish (e.g. [22]–[24]) and distinction between mixing and non-mixing stocks (e.g. [21], [25]–[27]). Attempts have also been made to use them for reconstructing horizontal and vertical migrations in marine fish (see [28] for a review). Yet this requires a thorough understanding of the links between otolith δ^18^O and environmental conditions, including temperature, salinity and water δ^18^O.

A major assumption underlying the use of otolith δ^18^O values to reconstruct temperature is that the isotopic fractionation between otolith aragonite and ambient water is constant between and within species. However, while otolith δ^18^O heterogeneity across waterδ^18^O-temperaturecombinations has led several authors to explore the possibility of differential fractionation equations, at least between species (e.g. [29]–[31]), this assumption has never been ground-truthed using free-swimming fish in their natural environment.

Physiological (i.e. "vital") effects, either kinetic or metabolic, have been shown to cause departure from equilibrium during oxygen fractionation in several biogenic carbonates (e.g. [32]–[35]), and have been repeatedly suspected during otolith formation (e.g. [30], [36], [37]), although this possibility has never been fully addressed. Experimental studies investigating the influence of "vital effects" on water-otolith δ^18^O fractionation have been few to date, all involving immature fish [31], [36], [38]–[43]. All concluded that otolith δ^18^O was driven mainly by ambient temperatures, largely independently of fish metabolism, especially when compared to δ^13^C signatures [36], [38], [43]. Yet, even though aquarium-based experiments allow for tank-based control of δ^18^O and reduction in inter-individual metabolic differences, δ^18^O heterogeneity across temperature-salinity combinations has consistently been observed, commonly ranging between 0.5 to 1.5 ‰ (e.g. [36], [38]–[41], [43]), equating to 2.3°C–6.1°C differences in temperature estimates (based on the equation of [2]). Cursory analysis has often related these findings to inter-individual differences in growth rate [38], even though they may equally reflect δ^18^O disequilibrium in the otoliths of some, if not all, the fish investigated. Physiological processes such as reproduction and growth rate have recently been shown to exert a major influence on the fractionation of trace metals between ambient water and otoliths, largely through modifications of ion-binding conditions in plasma [44]. Accordingly, the influence of vital effects on otolith δ^18^O signatures might have been largely overlooked to date. This raises the question as to the accuracy of climatic or geographic inferences drawn from otolith δ^18^O values, since free-ranging fish from genetically distinct stocks will experience greater environmental and metabolic variability.

In this study, we took advantage of concomitant environmental and migratory data gathered from a decade studying the spatial dynamics of mature female plaice (*Pleuronectes platessa* L.) in the North Sea using archival data storage tags [45], [46] to gain insight into otolith δ^18^O signature acquisition in wild fish populations. By comparing measured and predicted otolith δ^18^O values in three sub-stocks with discrete annual distributions, we explored the relationship between temperature, salinity and otolith δ^18^O values of mature, free-ranging fish in their natural environment. Two complementary approaches were applied to assess whether regional differences in physicochemical conditions in the offshore marine environment can confidently determine stock of origin, and to examine how accurately variations in otolith δ^18^O values reflect differences in ambient temperature. First, using modelled temperature and salinity data and the equation describing temperature-dependence of oxygen isotope fractionation during inorganic aragonite deposition [2] we predicted annual δ^18^O values over the full distributional ranges of our fish. Predicted values were then compared with the δ^18^O values measured in a subset of tagged fish with known temperature and migratory history to examine the potential interference of intra-specific variation in otolith seasonal growth on the accuracy of sub-stock discrimination. Second, we compared high-resolution seasonal measurements of otolith δ^18^O values with monthly otolith δ^18^O values predicted from the temperatures measured *in situ* by the tagged fish. Finally, we used two different fractionation equations to explore the potential physiological influences on oxygen isotope fractionation (i.e. ‘vital effects’). The results presented provide new insights into the accuracy of otolith δ^18^O values as a proxy for ambient thermal conditions in wild mature fish, with implications for studies using otolith δ^18^O values for stock discrimination or climate change applications.

## Material and Methods

The studies that contributed data to this project were conducted in International waters where no permit was required (in the North Sea between 51–58°N and 0–8°E). Dispensation for the landing of undersize fish (immature plaice) in the North Sea under contract MF0152 was obtained from the UK government (Defra). Other than dispensation to land immature fish (obtained from Defra under contract Mf0152, see above), no other specific permissions were required for our North Sea plaice-tagging work. Plaice is not an endangered or protected species. As stated above, no new data was collected as part of our study, rather existing data from previous contracts has been utilized, all of which have already been fully referenced in our manuscript. Plaice were captured from 30 minute beam trawls, and no animals were sacrificed as part of this study. All of the work that contributed to our study was subject to approval by CEFAS Animal Welfare and Ethical Review Committee.

### Environmental data and otolith collections

The otolith and environmental data used in the current study originate from a concerted programme to study the population dynamics of plaice in the North Sea from 1993 to 2000. In total 785 mature, predominantly female plaice were tagged with archival data storage tags (see [46] for more details), resulting in the eventual recovery of 194 individual environmental data records of between 2 and 512 days.

**Figure 1:**
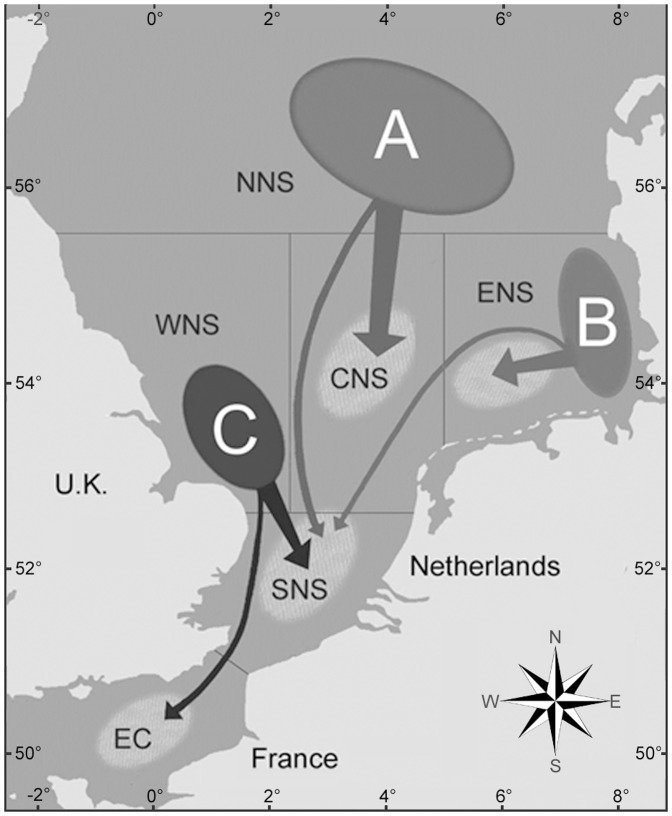
North Sea sub-stocks of plaice studied. The area covered by plaice migration between summer feeding aggregations (sub-stocks A, B, and C) and winter spawning grounds (light blue areas, [55]) was divided into 6 regions: the English Channel (EC: latitude <51.00°N), the Southern North Sea (SNS: latitude  = 51.00–52.49° N), the Western North Sea (WNS: latitude  = 52.50–55.49°N; longitude <2.50°E), the Central North Sea (CNS: latitude  = 52.50–55.49°N; longitude  = 2.50–4.99°E), the Eastern North Sea (ENS: latitude  = 52.50–55.49°N; longitude ≥5.00°E) and the Northern North Sea (NNS: latitude ≥55.50°N). Arrows show principal migration routes for each sub-stock [45].

The tags recorded ambient water temperatures (±0.2°C) and pressures (over the range 0–100 m, assuming seawater density of 1.025×10^3^), at 10-minute intervals throughout the period at liberty, providing detailed information on environmental conditions experienced, and allowing reconstruction of individual fish migration routes using the Tidal Location Method (TLM, [47]). Fish locations ( =  “geolocations”) were estimated when fish remained on the seabed for one or more tidal cycle. Pressure records were converted to depths (assuming 1 m = 1.46 psi), and the times of high water and accompanying tidal ranges measured by the resting fish were used to identify geolocation (see [45] for full details). “Best-fit” individual tracks were then reconstructed by fitting a piece-wise linear curve through the release position, any sequential geolocations and the recapture position (where provided). This allowed the generation of daily “positions” (grid references) for every fish and revealed the existence of three discrete summer feeding aggregations (sub-stocks, [46]), with predominantly southward migration of all fish to spawning areas in the Southern North Sea and Eastern English Channel during the winter (Fig. 1).

Among the 83 fish returned by professional fishermen with intact tags and otoliths, 24 individuals (8 per sub-stock) were selected to compare measured and predicted otolith δ^18^O values (Table 1). All were mature females released and recaptured between 1997 and 1999. This sub-sample was representative of the principal sub-stock specific migration patterns, and each data record allowed unequivocal identification of both spawning areas and feeding grounds for at least one annual cycle, sometimes two (Table 1).

**Table 1:** Details of fish analysed for annual otolith δ^18^O signatures of sub-stocks A (NNS), B (ENS) and C (WNS).

Sub-stock	ID	Recapture date	Size (cm)	Age (years)	DST record (start & end dates)	Breeding area and date (from DST records)	Year(s) analysed for δ^18^O
**A**	**A-1**	**May 99**	**44**	**14.3**	**512 days (Dec. 97 - April 99)**	**CNS (in January) ×** ***2***	**1997& 1998 (age 12**–**13)**
	**A-2**	**Jan. 99**	**40**	**5.9**	**398 days (Dec. 97 - Jan. 99)**	**CNS (in January)**	**1998 (age 5)**
	**A-3**	**July 99**	**40**	**12.5**	**386 days (Dec. 97 - Dec. 98)**	**CNS (in December)**	**1998 (age 11)**
	A-4	Dec. 98	40	8.8	365 days (Dec. 97 - Dec. 98)	CNS (in January)	1998 (age 8)
	**A-5**	**June 00**	**37**	**6.3**	**354 days (Feb. 99 - Feb. 00)**	**SNS (in February)**	**1999 (age 5)**
	A-6	Sept. 98	38	6.6	272 days (Dec. 97 - Aug. 98)	CNS (in February)	1997 (age 5)
	A-7	March 98	38	9.2	134 days (Oct. 97 - March 98)	CNS (in January)	1997 (age 8)
	A-8	March 98	39	8.1	110 days (Dec. 97 - March 98)	CNS (in January)	1997 (age 7)
**B**	**B-1**	**Dec. 98**	**48**	**7.9**	**382 days (Oct. 97 - Nov. 98)**	**SNS (in January) ×** ***2***	**1997& 1998 (age 6**–**7)**
	**B-2**	**Feb. 99**	**46**	**12.0**	**317 days (Oct. 97 - Aug. 98)**	**SNS (in January)**	**1998 (age 11)**
	**B-3**	**Jan. 99**	**41**	**7.9**	**262 days (Oct. 97 - July 98)**	**ENS (in January)**	**1997 (age 7)**
	**B-4**	**June 98**	**41**	**5.3**	**202 days (Nov. 97 - May 98)**	**ENS (in February)**	**1998 (age 5)**
	B-5	Oct. 99	38	7.7	194 days (Feb. - Aug. 99)	SNS (in February)	1999 (age 7)
	B-6	April 98	39	6.1	153 days (Nov. 97 - April 98)	ENS (in February)	1997 (age 5)
	B-7	Feb. 98	36	6.0	104 days (Nov. 97 – Feb. 98)	SNS (in January)	1997 (age 5)
	B-8	Feb. 98	40	7.0	97 days (Oct. 97 - Jan. 98)	ENS (in January)	1997 (age 6)
**C**	**C-1**	**April 99**	**52**	**9.1**	**411 days (Feb. 98 - April 99)**	**EC (in December) ×** ***2***	**1997& 1998 (age 7**–**8)**
	**C-2**	**Nov. 99**	**36**	**7.8**	**384 days (Oct.98 - Oct. 99)**	**EC (in January)**	**1999 (age 7)**
	**C-3**	**March 00**	**39**	**12.1**	**303 days (Feb. 98 - Dec. 99)**	**EC (in January)**	**1999 (age 11)**
	**C-4**	**Aug. 99**	**38**	**5.5**	**231 days (Oct. 98 - May 99)**	**SNS (in January)**	**1999 (age 5)**
	C-5	Oct. 99	41	10.7	223 days (Feb. - Sept. 99)	SNS (in January)	1999 (age 10)
	C-6	Sept. 98	44	10.6	221 days (Dec. 97 - July 98)	SNS (in December)	1998 (age 10)
	C-7	Sept. 99	41	9.6	182 days (Feb. - Aug. 98)	SNS (in January)	1998 (age 9)
	C-8	June 98	39	11.3	109 days (Feb.- May 98)	EC (in December)	1997 (age 10)

Bold indicates individuals selected for detailed analysis of intra-annual variations in δ^18^O. CNS: central North Sea; ENS: eastern North Sea; SNS: southern North Sea; EC: English Channel.

### Otolith preparation and analysis

All otoliths (paired sagittae), previously stored in paper envelopes, were cleaned in ultra-pure water and sonified for 5 minutes to remove organic surface debris. Additional loose material was removed by gently brushing the surface with a sterile toothbrush under reflected light (×50 magnification). Otoliths were then triple-rinsed in ultra-pure water and dried overnight in open acid-washed polypropylene vials stored in a clean vertical laminar flow workstation. Otoliths were embedded in clear epoxy resin within ethanol-washed aluminium moulds, and the resin dried for at least three days at 40°C before sectioning.

Although the paired otoliths exhibit differential growth from metamorphosis [48], this does not induce differences in seasonal oxygen isotope signature between them [43]. Therefore, to maximize data collection, both otoliths were used per fish. The left (symmetrical) otolith was cut twice (in the transverse then the frontal plane) and used for ageing (transverse section) and measuring annual otolith δ^18^O signature(s) deposited during the DST recording time (frontal section). The right (asymmetrical) otolith was used for high-resolution intra-annual δ^18^O analyses (frontal section), to maximize temporal resolution.

#### Fish ageing

Transverse otolith sections (∼500 µm thick) were mounted on glass slides using epoxy resin and ground down using 600 grit silicon carbide paper and ultra-pure water until the otolith core was exposed. Sections were then polished with 0.3 µm aluminium oxide paste and rinsed in ultra-pure water. Annual growth bands were identified and counted twice by two independent readers. Non-matching estimates were followed up with a third reading to reach a final decision. Age estimation applied a common notional birth date (after [13]) of February 1^rst^, based on peak plaice spawning times in the North Sea [49], [50]. Individual age (in years) was calculated by adding the annual fraction between the capture date and February 1^rst^ to the number of complete annual growth bands identified in the otolith.

#### Annual otolith δ^18^O measurements

Annual otolith δ^18^O values were measured for all individuals, for 1–2 years of life, depending on the recording time of the archival tags (Table 1), allowing the accumulation of nine annual δ^18^O values per sub-stock across the 1997–99 tagging period. Frontal sections (longest point from core to rostrum) approximately 500 µm thick were made from the left otoliths. Both sides were ground down to 300–400 µm using 600 grit silicon carbide paper and ultra-pure water until the otolith edge within the section was perpendicular to the section surface. Sections were then embedded in an epoxy mount, polished with 0.3 µm aluminium oxide paste, rinsed in ultra-pure water and photographed under reflected light. For each fish, one to two yearly powder samples (reflecting the total growth during archival tag data recording while the fish was at liberty) were collected from the otolith frontal section, using a computer-controlled micro-milling system (New Wave Research "MicroMill"). The seasonal growth marks were used to identify DST-recording periods from magnified images of the sections, and were digitized to provide navigational input to the instrument. One or two sequential layers of 250 µm depth, each comprising an entire annual growth band, were then milled from the distal edge (most recent growth) inwards. The corresponding powder samples (60–100 µg in weight) were collected separately and analysed at the Woods Hole Oceanographic Institution using a Finnigan MAT252 mass spectrometer system with a Kiel III carbonate device. All isotopic values were reported relative to the international carbonate standard VPDB, using the international standard delta notation:
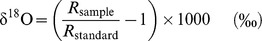



Where *R* is the ^18^O/^16^O ratio in the sample or standard. Analytical precision for δ^18^O values, based on the SD of daily analysis of NBS-19 carbonate standard, was ±0.07 ‰.

#### Seasonal otolith δ^18^O measurements using Secondary Ion Mass Spectrometry (SIMS)

For a subset of representative fish with data records >200 days (n = 4 per sub-stock, Table 1), high-resolution intra-annual measurements of otolith δ^18^O values were made over the tag-recording period. Otolith sections (∼500 µm thick) were ground down to 300–400 µm using 600 grit silicon carbide paper and ultra-pure water, ensuring that the otolith edge within the section was perpendicular to the section surface. The sections were embedded in an epoxy mount, polished with 0.3 µm aluminium oxide paste, rinsed in ultra-pure water and imaged under reflected light to record the growth band positions. Before analysis at the Edinburgh Ion Microprobe Facility (EIMF), mounts were gold-coated. Otolith δ^18^O values were measured on a CAMECA IMS 1270 ion microprobe using a ∼5 nA primary ^133^Cs^+^ beam. Ablations approximately 20 µm in diameter (equivalent to 1–3 months growth, depending on age and otolith growth rate) were executed at 30–40 µm intervals. Secondary ions were extracted at −10 kV, and ^16^O (∼3.0×10^9^ cps) and ^18^O (∼4.0×10^6^ cps) were monitored simultaneously on dual Faraday cups (L'2 and H'2). Each analysis involved a pre-sputtering time of 50 s, followed by automatic secondary beam and entrance slit centring and finally data collection in two blocks of 10 cycles. Otolith δ^18^O values are reported relative to VPDB. Mean external precision, based on the SD of daily analysis of an inorganic carbonate standard (University of Wisconsin Calcite standard UWC-1, [51]), was ±0.20 ‰.

### Predictions of otolith δ^18^O values

#### Sub-stock simulation of annual otolith δ^18^O values

Annual otolith δ^18^O values were predicted using the full range of environmental conditions likely to have been experienced by individuals of the three sub-stocks (Fig. 1) over the three year study period (1997–1999). Using all geolocations derived from the 194 tagged fish recaptured between 1993 and 2000 (n = 13,512), monthly distributions were summarized using grid-maps, showing the cells (0.5° latitude ×0.5° longitude) containing 80% of geolocations for each sub-stock. Average monthly seabed temperatures and salinities in 1997, 1998 and 1999 were generated for corresponding grid cells using the General Estuarine Transport Model (GETM), developed and validated for realistic three-dimensional simulations of temperature and salinity in the North Sea [52]. The model domain extends from a boundary in the western English Channel (−5°E) into the North Sea with an eastern boundary in the Baltic (16°E) and then northwards as far as the Shetland Isles (60°N) at a resolution of ∼6 nm and with 25 terrain-following vertical levels. Meteorological forcing in the model for the 3 years studied was derived from the European Centre for Medium-range Weather Forecasting ERA datasets. Tidal boundaries were calculated from Topex-Poseidon satellite altimetry, and temperature and salinity boundary conditions were taken from the climatologic predictions of the POLCOMS S12 model (http://cobs.pol.ac.uk/modl/metfcst/POLCOMS DOCUMENTATION).

For each sub-stock, monthly GETM temperature and salinity estimates for each grid cell and year were used to predict corresponding otolith δ^18^O values. For this, oxygen isotope ratios of ambient water (δ^18^O_w_) were derived from salinity (*S*) estimates using the equation of [53] for the North Sea:
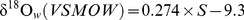
(1)


Then converted into δ^18^O_w_ (*VPDB*) using the equation of [54]:
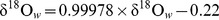
(2)


Finally, the corresponding temperature estimates (T, in K) were incorporated in order to predict otolith δ^18^O values (δ^18^O_o_) using the theoretical equation for inorganic aragonite deposition [2]:
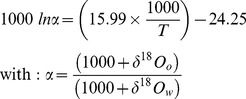
(3)


The resulting monthly maps of otolith δ^18^O values were used to simulate the sub-stock specific range of annual otolith δ^18^O signatures. For this, we generated 10,000 annual migration paths, using a “stock”-constrained random walk simulation (within each stock, individual "fish" were free to move randomly each month to any adjacent cell within the sub-stock specific range), so all simulated paths followed stock-specific seasonal migration patterns. Then, to account for the influence of intra-specific variations in seasonal otolith growth on the annual δ^18^O signatures for each sub-stock, three alternative plausible scenarios for otolith annual growth were applied to differentially weight the monthly values for each simulated migration path.

Seasonality of opaque-translucent otolith banding fluctuates according to fish age and latitude in North Sea plaice [55], implying potential for geographic and individual variations in monthly otolith growth rates. The opaque zone is mainly accreted during April to September, and the hyaline (or translucent) zone from October to March [55]. In the otoliths from our study, the opaque zone occupied 50–60% of the total width of the annual growth band, compared with 40–50% for the hyaline zone. In the North Sea, plaice spawning occurs from the end of December to the beginning of April [56]. Females are in spawning condition for at least 5 weeks, during which feeding ceases [49], as spawning and feeding are mutually exclusive due to limited metabolic scope that does not allow oxygen supply for both activities [57]. Metabolic rate during spawning can decrease to 9.1 kJ per day, i.e. 60% of the standard metabolic rate, estimated at 15 kJ per day [57]. Based on these physiological data, we considered the three possible scenarios for otolith annual growth, considering an equal duration of 6 months for both opaque (April-September) and hyaline zone (October-March) depositions:

- OG1: otolith growth is constant throughout the year, i.e. the width of opaque and hyaline zones are identical (equal weighting of 100% given to all months),
- OG2: otolith growth varies through the year, with the width of the opaque zone representing 60% of annual growth and the hyaline zone representing 40% (weighting differs among months: April to September  = 100% and October to March  = 67%),
- OG3: otolith growth varies through the year, with the width of the opaque zone representing 60% of annual growth and the hyaline zone representing 40% but growth being 40% lower during the three breeding months (different weighting among months: April to September  = 100%, October to December  = 83% and January to March  = 50%).

The 10,000 simulated random annual migration paths per sub-stock, weighted to each of the three growth scenarios, produced 30,000 plausible annual δ^18^O values per sub-stock. This provided a robust platform of predicted δ^18^O values with which to compare the measured values in our tagged fish.

#### Prediction of otolith δ^18^O values during DST recording time

For the 12 individuals selected for SIMS analysis (N = 4 per sub-stock, Table 1), daily otolith δ^18^O values between release and recapture were predicted using the corresponding tag-recorded temperatures. Equivalent ambient salinities for the individual daily geolocations were extracted from the CEFAS database for North Sea bottom salinity, where available (16%), or were predicted using the GETM. Water salinities were converted into δ^18^O_w_ (*VPDB*) values using equations (1) and (2). Daily otolith δ^18^O values (δ^18^O_o_) were predicted from tag-recorded temperatures using both equation (3) from [2] and an equation recently obtained for juvenile plaice reared under controlled conditions [43]:
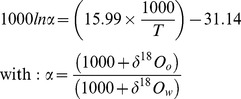
(4)


Depending on the size and age of the fish, our high-resolution measurements of otolith δ^18^O represented (at best) monthly resolution. Therefore, individual monthly averages were calculated from predicted daily δ^18^O estimates, and were compared with corresponding measured intra-annual δ^18^O values. Intra-annual otolith growth was assumed to be non-linear, and to vary between individuals. To calibrate distances within individual otoliths to calendar dates, the time axis on the measured profile was thus adjusted (using Analyseries 2.0 [57]) to achieve the best match in the position of inflection points between the predicted and observed time series of δ^18^O values.

### Data analysis

Inter-annual differences in the salinity, temperature and otolith δ^18^O values expected over the full distributional area of all three sub-stocks (53,655 values per year in each case, derived from average daily model predictions for the 147 cells of the global distribution map) were tested separately by 1-way (year) fixed-effects, unbalanced analyses of variance (ANOVAs). Tests for data normality, homoscedasticity and independence of residuals were achieved using Shapiro-Wilk normality tests, studentized Breusch-Pagan tests and Durbin-Watson tests of residuals, respectively. Post-hoc comparisons were performed using the Tukey test of multiple means comparisons. Similarly, differences in the annual environmental exposure (salinity, temperature) and otolith δ^18^O values (both measured and predicted) of the three sub-stocks were tested separately by 1-way (sub-stock) fixed effects, unbalanced ANOVAs and Tukey tests.

Sub-stock discrimination accuracy from otolith annual δ^18^O values was evaluated using linear discriminant analyses (LDA function from the R package MASS). To avoid circularity when the same values are used to build and test the model, "leave-one-out" procedure was used to train the LDA with otolith δ^18^O predicted values in each case (N = 30,000 per sub-stock for the grouped otolith growth scenarios and 10,000 per sub-stock when selecting only the best fit scenario). Overall correctness in sub-stock prediction was then assessed using the actual measured otolith annual δ^18^O values (N = 27, nine per sub-stock).

Lastly, differences between measured and predicted intra-annual otolith δ^18^O values were tested separately for each sub-stock, by comparing both minimum and maximum values (N≥4) in each case using non-parametric unilateral Wilcoxon tests for paired-samples.

All statistical analyses and simulations were performed in R (R Development Core Team, 2011).

## Results

### Predicted otolith δ^18^O values

Over the full distributional range experienced by our tagged plaice, predicted bottom temperatures did not vary significantly between 1997, 1998 and 1999 (F = 0.84; P = 0.432; df = 2), fluctuating around an annual mean (± SD) of 10.41°C±3.85°C. Corresponding predicted bottom salinities did vary significantly by year (F = 20.01; P<0.001; df = 2), with a slightly higher mean annual value in 1998 (34.66±0.34) than in 1997 (34.57±0.22) and 1999 (34.55±0.25). However, this variation (ca. 0.11) did not significantly influence δ^18^O values, and the constancy in among-year temperature profiles ensured inter-annual stability (F = 1.46; P = 0.235; df = 2) in the predicted annual otolith δ^18^O values across the study area over the period studied (mean: 1.89±0.98 ‰). The three years' environmental data were therefore pooled to describe sub-stock specific temperature and salinity conditions and the calculation of predicted δ^18^O values.

**Figure 2:**
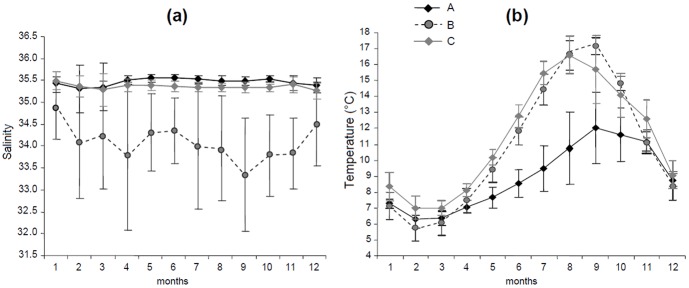
Fish environmental experience. Annual cycle of mean (± SD) salinities (a) and temperatures (b) predicted for the 3 plaice sub-stocks (A, B and C) in the North Sea, based on all the individual locations observed for each month and corresponding environmental conditions derived from the General Estuarine Transport Model for the period 1997–99.

The reconstructed environmental conditions experienced by the three sub-stocks differed markedly (Fig. 2), as predicted from their geographically distinct summer feeding locations and winter migration routes (Fig. 1). Annual temperature profiles were comparable for sub-stocks B and C, with minima around 6.5°C in February-March and maxima around 16.5°C in August-September. By contrast, temperatures experienced by sub-stock A never exceeded 12°C. Annual salinity profiles were similar for sub-stocks A and C, with relatively constant salinities throughout the year (34.8 - 35.1). This contrasted with sub-stock B, for which salinities were <34.5 and more variable irrespective of the month (Fig. 2).

**Figure 3:**
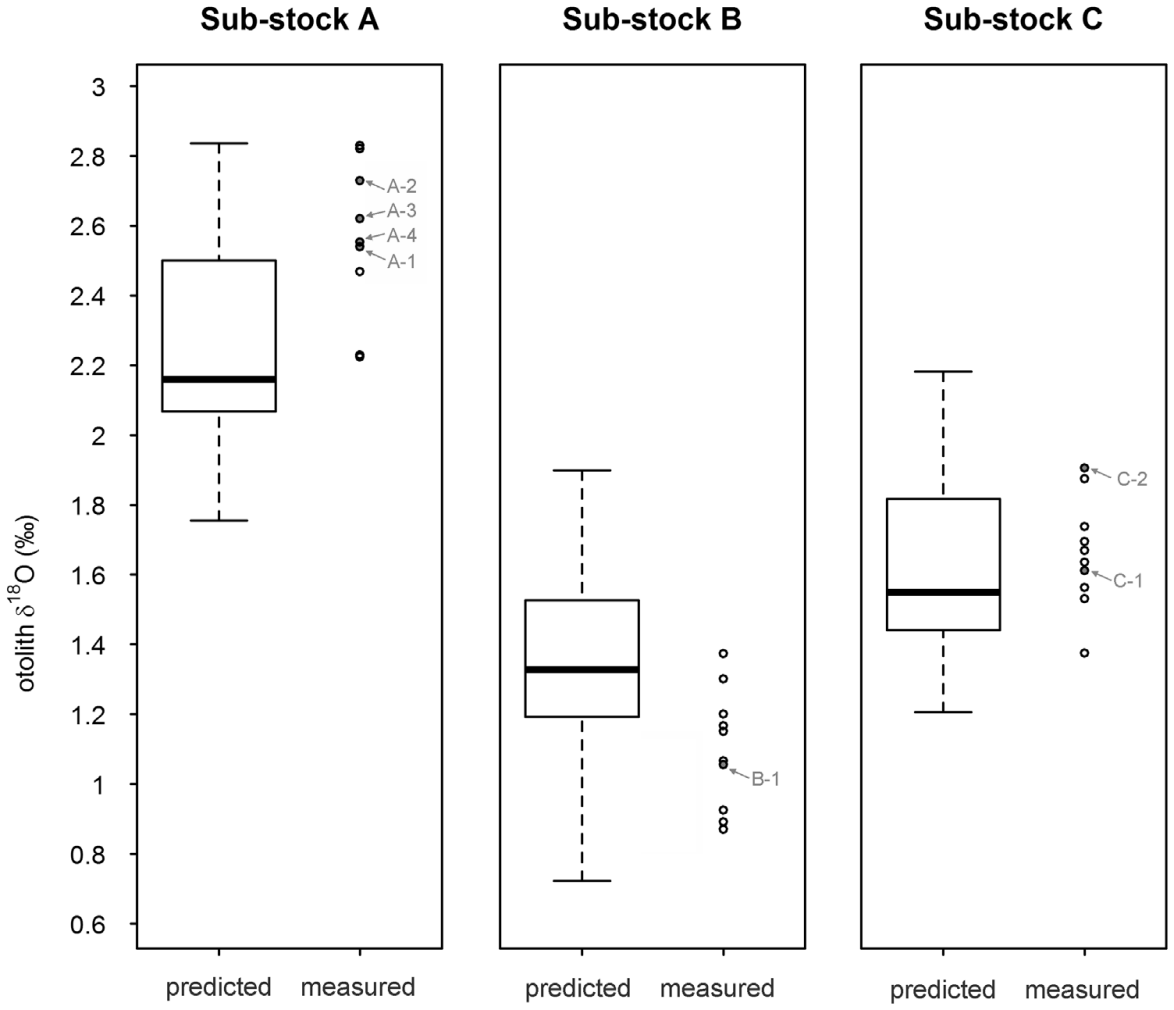
Overall annual otolith δ^18^O predictions per sub-stock. Predicted annual δ^18^O values (in white), obtained by applying all 3 plausible otolith growth scenarios to the monthly δ^18^O derived from the stock-specific temperature and salinity conditions experienced (30,000 simulations), and corresponding measured annual δ^18^O values (circles) per sub-stock. In each boxplot, the box includes 50% of the data and the whiskers 100%, with the bold line within the box indicating the median. For each sub-stock, the individual otolith annual δ^18^O values of the fish returned with a full annual DST record (c.f. Table 1) are indicated in grey.

As a result, otolith δ^18^O values were predicted to vary by month and by sub-stock (Table 2). For all sub-stocks, intra-annual patterns in predicted δ^18^O values predominantly followed the annual cycle in bottom temperature (Fig. 2). The lowest average values (−0.06 to 1.69 ‰) were consistently predicted for August and September (the warmest months) and the highest (2.40 to 2.64 ‰) for February and March (the coldest months). Despite some overlap in monthly stock-specific δ^18^O maxima and minima, the average monthly predictions remained distinct throughout the year. Consequently, no major overlap in annual sub-stock otolith δ^18^O values was predicted, even when considering contrasting scenarios for otolith growth (Fig. 3). Predicted annual ranges of otolith δ^18^O values differed between sub-stocks (F = 135,117; P<0.001; df = 2), with significantly lower values (P<0.001) in sub-stock B (1.10±0.17 ‰), and higher values (P<0.001) in sub-stock A (2.58±0.23 ‰), than in sub-stock C (1.66±0.16 ‰).

**Table 2:** Monthly otolith δ^18^O values (in ‰) predicted for the 3 sub-stocks over the 3-year study-period (1997–99).

Month	Sub-stock A	Sub-stock B	Sub-stock C
**1**	2.46 *(2.25−2.59)*	2.35 *(2.04−2.59)*	2.22 *(2.00−2.44)*
**2**	2.64 *(2.34−2.75)*	2.45 *(1.81−2.70)*	2.51 *(2.37−2.65)*
**3**	2.64 *(2.05−2.79)*	2.40 *(1.79−2.71)*	2.48 *(2.15−2.63)*
**4**	2.52 *(2.31−2.64)*	1.96 *(0.54−2.47)*	2.26 *(2.06−2.36)*
**5**	2.40 *(2.05−2.56)*	1.67 *(1.13−2.07)*	1.79 *(1.62−1.99)*
**6**	2.20 *(1.83−2.42)*	1.13 *(0.76−1.60)*	1.21 *(0.98−1.39)*
**7**	1.98 *(1.48−2.43)*	0.47 *(-0.39−1.11)*	0.63 *(0.42−0.91)*
**8**	1.69 *(0.76−2.32)*	-0.06 *(-0.77−0.21)*	0.38 *(0.03−0.83)*
**9**	1.41 *(0.77−2.06)*	-0.32 *(-0.73−0.04)*	0.57 *(0.16−1.61)*
**10**	1.52 *(0.93−2.08)*	0.36 *(0.06−0.65)*	0.91 *(0.55−1.77)*
**11**	1.58 *(1.32−1.85)*	1.15 *(0.91−1.30)*	1.26 *(1.00−1.52)*
**12**	2.11 *(1.94−2.35)*	1.93 *(1.73−2.04)*	2.00 *(1.64−2.10)*

For each sub-stock and month, the range (minimum - maximum) of values predicted for all the grid cells occupied is shown in italics beside the corresponding mean.

### Correspondence between predicted and measured annual otolith δ^18^O

Measured annual δ^18^O values all fell within predicted ranges (Fig. 3), and as predicted, differed between sub-stocks (F = 158.3; P<0.001; df = 2,). Annual δ^18^O values were significantly lower (P<0.001) in sub-stock B (1.35±0.21 ‰), and higher (P<0.001) in sub-stock A (2.25±0.24 ‰), than in sub-stock C (1.61±0.21 ‰). When trained with the grouped otolith growth scenarios' δ^18^O predictions, the LDA procedure had an overall discrimination accuracy of 99.76% using the cross-validation procedure. For fish of known origin, 96.70% correct sub-stock identification was attained. The only misclassification belonged to female C-6 (Table 1), wrongly assigned to sub-stock B.

**Figure 4:**
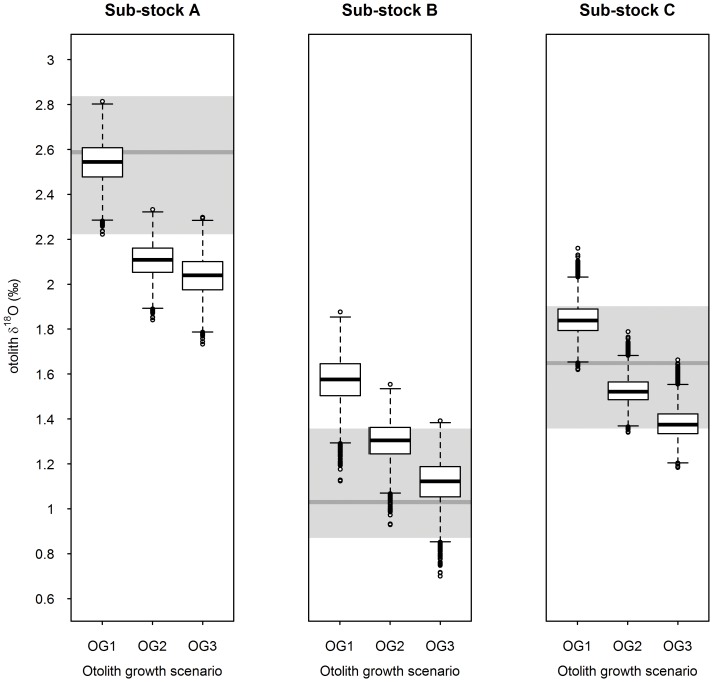
Annual otolith δ^18^O predictions per growth scenario for the 3 sub-stocks. Predicted annual δ^18^O values (in white) were obtained by individually applying each of the 3 plausible otolith growth scenarios (OG1, OG2 and OG3) to monthly δ^18^O derived from the stock-specific temperature and salinity conditions experienced (10,000 simulations per scenario and sub-stock). In each case, the white box includes 50% of the data and the whiskers 99%, with the bold line within the box indicating the median and the circles the outlier values. For comparison, the full range of observed annual δ^18^O values is represented for each sub-stock (in grey, the bold line indicating the median).

However, differences in measured annual otolith δ^18^O values between sub-stocks were greater than predicted from their temperature and salinity profiles, with mismatches between measured annual and predicted δ^18^O values, notably for sub-stocks A and B (Fig. 3). This may be partly explained by differences in annual otolith growth patterns among sub-stocks since OG1, OG2, and OG3 each produced different stock-specific annual estimates (Fig. 4). If so, our results suggest that fish predominantly follow OG1 in sub-stock A, OG3 in sub-stock B, and OG2 in sub-stock C.

Training the LDA using only predicted δ^18^O values from the best matching growth scenario for each sub-stock (i.e. OG1 for sub-stock A, OG3 for sub-stock B and OG2 for sub-stock C) raised overall discrimination accuracy to >99.99% using the cross-validation procedure (Fig. 4), but did not, improve identification in the 27 measured annual δ^18^O values (96.70%), due to B-3's incorrect assignment to sub-stock C (Table 1). Furthermore, half of the measured annual values fell outside the 75% predictions irrespective of the sub-stock (Fig. 4). This provides further evidence of a slight mismatch between measured and predicted values for all three sub-stocks. For sub-stock C this could arise as a result of OG1 growth rather than OG2 in some individuals tested. However this would not explain the higher and lower than predicted δ^18^O values observed for sub-stock A and B. Comparison of measured and predicted annual otolith δ^18^O values in the seven fish returned with full annual DST records (Table 1) confirmed this pattern. Measured annual δ^18^O values were slightly, yet consistently, higher (of 0.09‰ in A-1, 0.31‰ in A-2, 0.22‰ in A-3 and 0.11‰ in A-4) than those predicted from concomitant *in situ* temperature measurements in sub-stock A, irrespective of the otolith growth scenario used. Conversely, measured annual δ^18^O was >0.13‰ lower than predicted in B-3. For sub-stock C, the difference between measured and predicted annual δ^18^O values was below analytical error (<0.07‰) for both C-1 and C-2, when using OG2 and OG1, respectively.

### Correspondence between predicted and measured high-resolution otolith δ^18^O values

**Figure 5:**
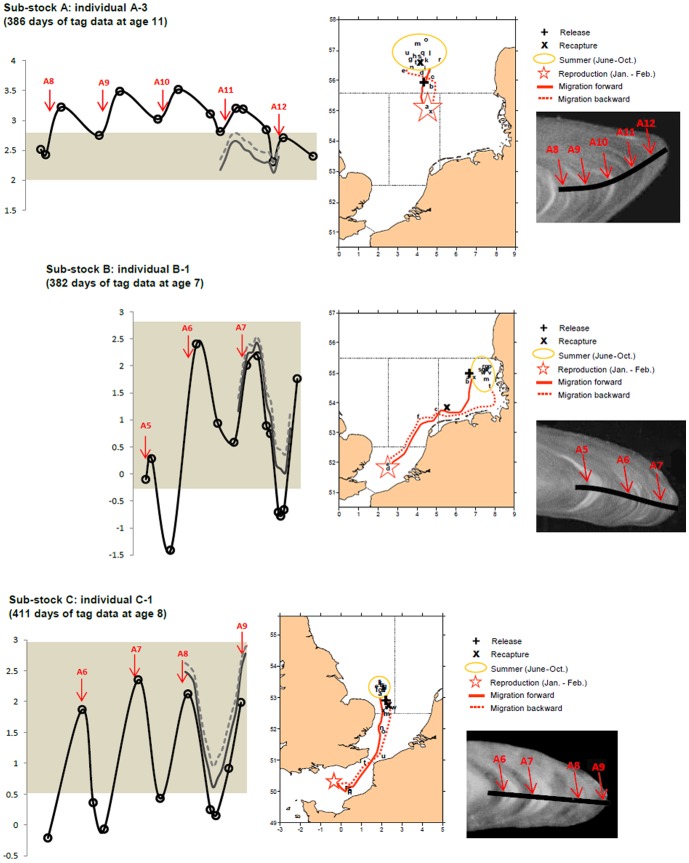
Intra-annual patterns of otolith δ^18^O variation. Multi-annual seasonal variations of adult otolith δ^18^O records (black line with spots, graph) measured with Secondary Ion Mass Spectrometry (SIMS) in 3 individuals representative of their sub-stock (A, B or C - see Fig. 1), shown with matching δ^18^O values predicted from *in situ* tag data using the equation of Kim *et al*. 2007 for inorganic aragonite (plain grey line, graph) or that of Geffen 2012 for juvenile plaice (dotted grey line, graph). The shaded boxes (graph) indicate fish-specific range of daily tag-predicted δ^18^O values during their time at liberty. The red arrows indicate the successive positions of otolith annuli both on the graphs and on the corresponding photographs, i.e. the detailed views of the longitudinal sections analysed with SIMS (black line). Maps show the reconstructed movements of the fish during tag recording time, with the black letters indicating successive geolocations between fish release and recapture in each case.

The temporal patterns of high-resolution otolith δ^18^O values measured using SIMS generally corresponded to those predicted from concomitant monthly records of environmental conditions (Fig. 5). However, some δ^18^O values fell outside the predicted range irrespective of the equation applied (i.e. that for inorganic aragonite [2] or that for juvenile plaice [43]), confirming the slight dissimilarities already observed between predicted and measured annual δ^18^O values.

**Figure 6:**
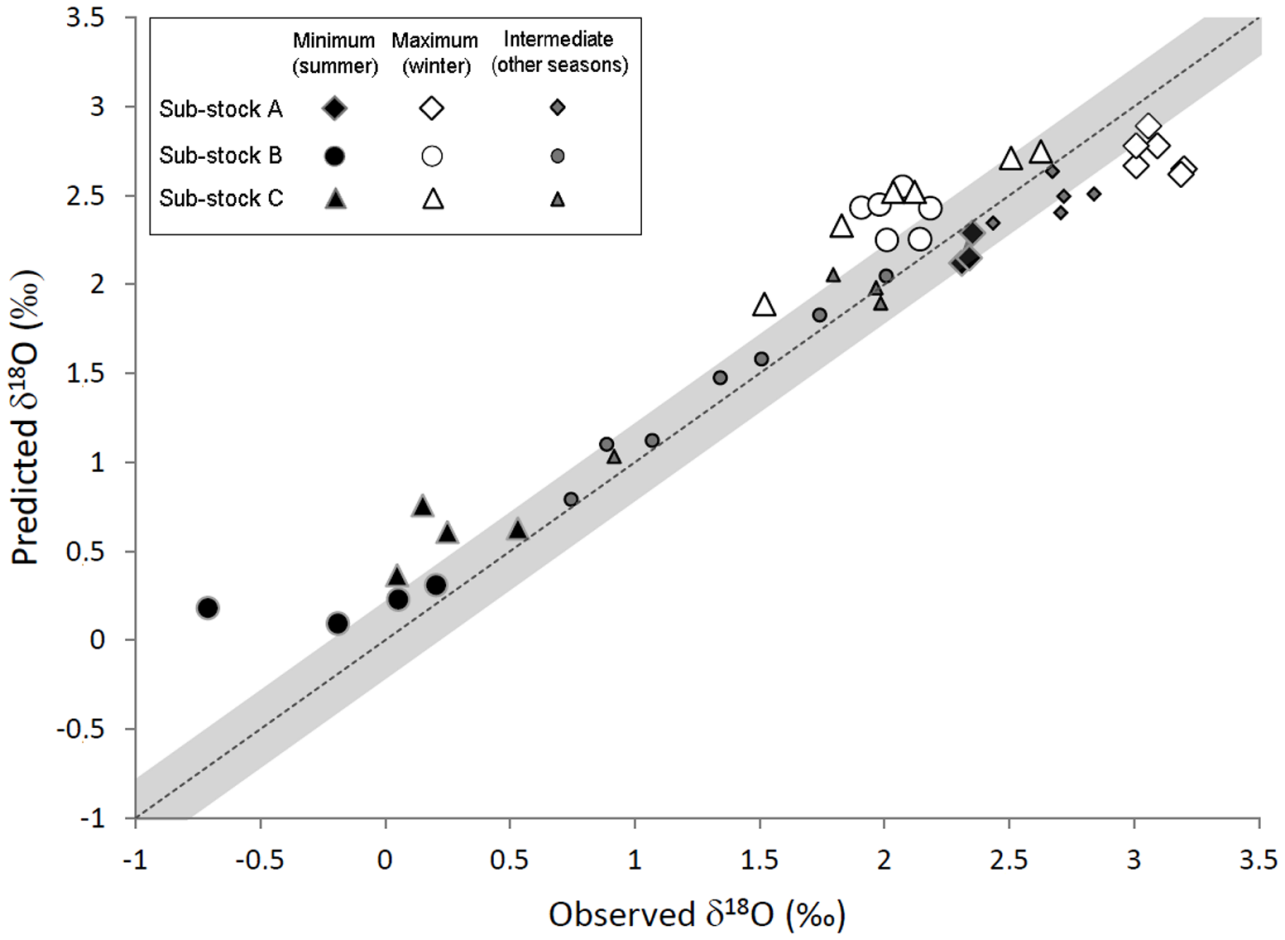
Sub-stock specific vital effects? Measured *vs*. predicted intra-annual otolith δ^18^O values obtained for the 4 fish per sub-stock analysed with SIMS. In each case, intra-annual otolith δ^18^O measurements made during DST recording time were matched with best corresponding monthly δ^18^O values predicted from concomitant *in situ* tag temperature records using the equation from Kim et al. (2007). The grey area around the 1∶1 line represents the analytical error using SIMS. NB: the number of measurements differs between sub-stocks due to inter-individual differences in tag recording times. For all individuals, minimum predicted monthly δ^18^O values were consistently observed in the summer (in July, August or September) and maximum ones in the winter (in February or March).

When using the equation for inorganic aragonite [2], the differences between measured and predicted δ^18^O values were generally small (Fig. 5 and 6), with an average of 0.28±0.21‰ for the three sub-stocks combined, which is similar to the 0.2‰ measurement error of the SIMS analyses. Measured and predicted δ^18^O values exhibited a strong relationship, close to the 1∶1 line (Fig. 6). However, the differences between them exceeded 0.5‰ in several individuals, irrespective of sub-stock (Fig. 6). Moreover, the directionality of the differences between the predicted and observed values varied among, but was consistent within sub-stocks. Measured δ^18^O values were significantly and consistently higher than predicted for individuals in sub-stock A, while individuals from sub-stocks B and C exhibited measured δ^18^O values significantly lower than predicted during the coldest (February, March, April) and warmest (July, August, September) months of the year (Fig. 5 and Table 3). These differences were consistent with the trends observed in annual δ^18^O values when annual otolith growth predominantly followed OG1 in sub-stock C (Fig. 4). Visual examination of the sub-stock C otoliths seemed to confirm this result, as the opaque zone was not particularly wider than the hyaline zone in several of the specimens analysed (e.g. Fig. 5).

**Table 3:** Average (±SE) differences between measured and predicted extreme δ^18^O values (in ‰) obtained for the 3 sub-stocks when using either the fractionation equation for inorganic aragonite [2] or that for juvenile plaice reared under controlled conditions [43].

	Obs. – pred. Inorganic aragonite	Obs. – pred. Juvenile plaice
***Sub-stock A***		
**Minimum δ^18^O**	0.21±0.03 (*)	0.01±0.03 (ns)
**Maximum δ^18^O**	0.33±0.03 (***)	0.23±0.02 (***)
***Sub-stock B***		
**Minimum δ^18^O**	−0.36±0.09 (*)	−0.71±0.09 (*)
**Maximum δ^18^O**	−0.34±0.03 (**)	−0.47±0.03 (**)
***Sub-stock C***		
**Minimum δ^18^O**	−0.38±0.03 (**)	−0.74±0.03 (**)
**Maximum δ^18^O**	−0.39±0.03 (**)	−0.52±0.03 (**)

Significant differences, based on unilateral Wilcoxon tests for paired samples, are shown at *P*<0.05 (*), *P*<0.01 (**) or *P*<0.001 (***).

**Figure 7:**
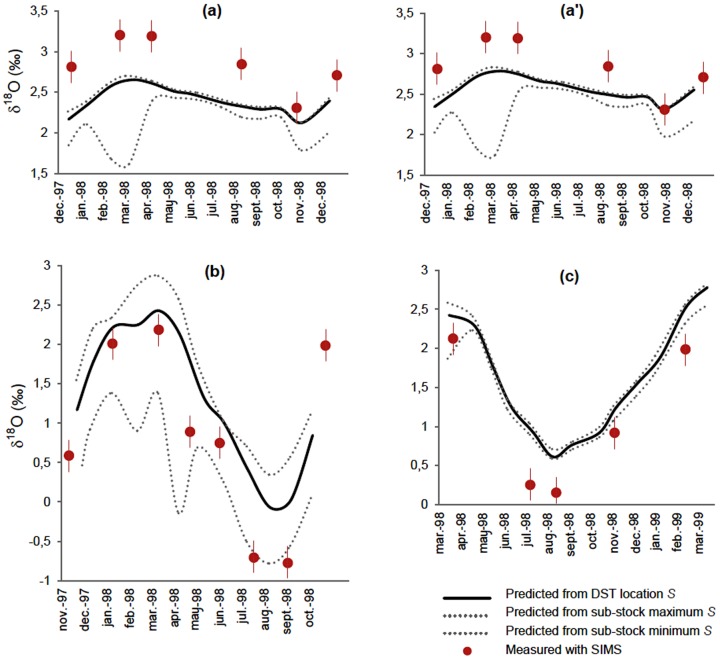
Uncertainty around salinity estimates do not explain δ^18^O offsets. Potential errors due to salinity estimation from fish geolocation with GETM on the predictions of monthly δ^18^O values for sub-stocks A (a), B (b) and C (c), when using the equation for inorganic aragonite (Kim et al. 2007). For sub-stock A, predictions using the equation for juvenile plaice (Geffen 2012) are provided for comparison (a'). Spots indicate successive intra-annual otolith δ^18^O values (with vertical bars showing analytic error with SIMS) during DST recording time for a representative fish per sub-stock (cf. Fig. 5). Matching values predicted from *in situ* tag temperature data, combined either with corresponding individual daily GETM salinity estimates (black solid line), or with the minimum and maximum daily salinity GETM estimates obtained monthly over the full 3-year distribution range of the sub-stock (grey dotted lines).

Predicting otolith δ^18^O values using the juvenile plaice fractionation equation [43] instead of that for inorganic aragonite [2] did not reduce the difference between predicted and measured δ^18^O values, with an average divergence of 0.45±0.30‰ for the three sub-stocks combined (Fig. 7). The difference between predicted and measured δ^18^O values for sub-stock A was reduced, especially for minimum values corresponding to the warmest months. However differences for sub-stocks B and C were amplified, both for minimum and maximum annual values (Table 3), which is the opposite trend to what one would expect if the differences were an artefact of signal attenuation and sampling resolution.

## Discussion

To date, studies focusing on the links between otolith δ^18^O and environmental conditions have mainly used captive juvenile fish in controlled tank experiments [31], [36], [39]–[43]. A small number of studies have examined controls on otolith δ^18^O *in situ* (e.g. [6], [16], [25], [30], [38], [58], [59]), but were limited in their findings due to difficulties in obtaining accurate positional and environmental data over appropriate temporal and geographical scales. In the current study, *in situ* temperature measurements, high resolution salinity estimates and individual migration pathways have for the first time been matched with concomitant otolith δ^18^O records in wild marine fish from distinct sub-stocks, providing useful insights into natural variations in otolith δ^18^O records of environmental conditions at sea and the value of this natural proxy for stock discrimination and seawater temperature reconstruction.

### Otolith δ^18^O values as "natural tags"

The release and recapture of hundreds of electronic archival-tagged plaice has revealed important insights into plaice spatial dynamics [60], and has here provided a unique platform to test whether otolith δ^18^O values can be used to derive equivalent positional data. Based on >13,000 geolocations from >190 individual fish, simulated temperatures and salinities over a three year period predicted significant and consistent differences in annual otolith δ^18^O profiles between the three plaice sub-stocks studied. The high accuracy (>96%) in sub-stock assignment using both measured and predicted annual δ^18^O values confirmed the utility of otolith δ^18^O values as a natural tag of stock origin (e.g. [24], [26], [27], [58]). Moreover, the marked differences in summer otolith δ^18^O values between sub-stocks (Table 2) demonstrated that, providing the ambient environment is sufficiently variable in temperature or salinity, δ^18^O signatures can allow broad-scale geolocation on a finer spatio-temporal scale than is currently applied in the management of most offshore fisheries [61]. In the North Sea, this offers the opportunity to map meta-population dynamics through stock identification of fish mixing on winter spawning grounds [62].

At a finer scale however, our results suggest that this approach to movement reconstruction should be applied with some caution, even in fully marine environments. As stated by many authors (e.g. [6], [27], [42]), oxygen isotope incorporation into fish otoliths is temperature-dependent, yet largely driven by water δ^18^O values, which in turn vary with salinity. Because ocean salinity can fluctuate locally and regionally, especially in the coastal zone [3], this can affect otolith δ^18^O values, and not only for species migrating between brackish or hypersaline habitats [14], [22], [63]. As shown here, even limited marine salinity variations (e.g. from 33 to 35) can generate pronounced differences in otolith δ^18^O values among fish experiencing similar temperatures during the year (e.g. sub-stock B *vs*. C). Except for active vertical movement during migration and spawning, the tagged plaice in the current study were largely at, or close to, the seabed [64]. As such, baseline knowledge of bottom temperature and salinity could generate δ^18^O "isoscapes" [65] as a predictor of potential marker efficacy for this species. However, vertical migrations could bias δ^18^O-based estimates of individual geolocation in other fish species due to vertical gradients in water temperature and salinity [53], [66].

Our results also demonstrate the importance of intraspecific variations in otolith growth in the generation of annual otolith δ^18^O values in the wild. The differences in measured annual δ^18^O ranges among sub-stocks using the fractionation equation for inorganic aragonite [2] were consistently greater than predicted when assuming similar otolith growth (i.e. under any of the three growth scenarios), suggesting inter-population variations in otolith deposition rates and/or the occurrence of vital effects (see below). Sub-stock and season-specific weighting of monthly δ^18^O values improved our predictions, but these latter still did not perfectly match measured annual δ^18^O values. The timing of otolith edge deposition in North Sea plaice varies with area, fish age and year [55]. Therefore, errors in the coupling of otolith growth bands with corresponding DST records cannot be fully excluded. Similarly, otolith growth may have followed different trajectories to the scenarios applied for annual δ^18^O predictions. However this does not explain the divergence between measured and predicted seasonal δ^18^O values observed in individual fish (e.g. Fig. 7).

For sub-stocks A and C at least, measured otolith δ^18^O values diverged from predicted, even when using *in situ* temperature records and accounting for potential errors in fish positioning and salinity modelling (Fig. 7). This possibly reflects departure from equilibrium during oxygen isotope fractionation in North Sea plaice otoliths, at least for certain times of the year. If so, our results furthermore suggest that the nature of this departure might differ among sub-stocks. Ideally, multi-stock validation studies across a wide range of ontogenies are required to establish a more comprehensive understanding of the relationship between fish metabolism and otolith δ^18^O.

### Possible sources of error in δ^18^O predictions

In the current study, we used measured and modelled environmental data to predict otolith δ^18^O values from estimated fish position. We are confident that our approach generally produced robust predictions of otolith δ^18^O values, both for individual fish and for sub-stocks. However, in generating unique *in situ* validation measures, we acknowledge two main inherent and unavoidable potential sources of error.

The first of these involves estimating fish location (geolocation) and the estimation of corresponding environmental data when no *in situ* measurements were available. The Tidal Location Method (TLM) is estimated to be mainly accurate to within 10 to 40 km according to time and location [47]. When combined with GETM's 20 km model grid cells of the GETM, this could attenuate localized temperature and salinity variations experienced by individual fish, resulting in inaccuracies in environmental predictions. However, GETM has proven to be extremely powerful at reproducing three-dimensional patterns of temperature and salinity [52], and the salinity range is very narrow (34.7–35.4) in most offshore areas of the North Sea [53]. Therefore, these two sources of error, even when combined, would only have limited impact (<0.2 ‰) on the majority of monthly otolith δ^18^O predictions (Figure 7). Exceptions could include the few individuals migrating to the localized areas of the central (at approximately 3°E, 57°N) or the eastern North Sea (close to the Dutch coast) penetrated by fresh waters from the Baltic Sea, and the Elbe and Rhine Rivers [53]. This only has potential to affect individuals from sub-stocks A and C during spawning migrations, but could potentially affect individuals from sub-stock B all year round (Figure 2a and 7b).

The second main potential bias in our δ^18^O predictions lies in the estimation of water δ^18^O from salinity. The equation we used [53] has been developed specifically for the North Sea, based on δ^18^O measurements from surface and bottom water collected over a 3-year period over the entire North Sea basin, and is in agreement with all the similar equations or δ^18^O measurements previously made in this area (e.g. [67], [68]). We are confident therefore, that our dataset is largely accurate. The δ^18^O salinity relationship can however be affected by localised inputs from the Rhine and Elbe Rivers [53]. As a result, any errors in our estimations will, again, be most pertinent to sub-stock B.

### Otolith δ^18^O values as temperature proxies

The first major requirement for temperature assessment using carbonate δ^18^O values is constancy in the isotopic composition of the surrounding seawater [69]. Few authors have evaluated the potential error in temperature estimations from otolith δ^18^O values due to salinity variations within their study area [6], [9], and this factor is often overlooked. Offshore salinity is generally assumed to be constant, both spatially and temporally, with a mean oceanic water δ^18^O value applied to all temperature back-calculations (e.g. [8], [14], [70]). Yet, even in offshore environments, water salinity and δ^18^O are never fully constant [53], [66]. Due to water stratification, vertical movements of fish are likely to exacerbate these variations [12]. Salinity will have greatest influences on water δ^18^O values at high latitudes and in coastal seas with high altitude catchments [28]. However, our results emphasise the influence of even small fluctuations in salinity, whereby salinity changes of just 1 sufficiently modified the water isotopic value in our study area to bias local temperature estimates by 1.3°C. Depending on the equation used to derive water δ^18^O values from ambient salinities, this difference could increase to 2.5°C, e.g. when using the North Atlantic equation [71]. Where ever possible, accurate salinity estimates should therefore be used when reconstructing water temperature using otolith δ^18^O values. Ideally, direct measures of water δ^18^O would be used, since pH variations can also modify water isotopic values [72]. This is not the case in the present day North Sea, where surface and bottom water δ^18^O measurements vary consistently with salinity throughout the basin [53]. However pH in other modern oceans can vary from 7.6 to 8.4 [73], potentially shifting water δ^18^O_VSMOW_ by ∼1‰ at 25°C [72]. More substantial variations in ocean pH have occurred over geological timescales, potentially biasing paleotemperature reconstructions by several °C [72].

The second major prerequisite in temperature reconstructions using otolith δ^18^O values is that deposition of oxygen isotopes in otoliths is in equilibrium with ambient δ^18^O, or that any departure from equilibrium is at least predictable [42]. To date, relatively few studies have investigated the temperature-δ^18^O fractionation relationship for fish otoliths, either under controlled aquarium conditions [31], [39]–[43], [74] or in the wild [6], [25], [30], [38], [58], [75], [76]. Of these few studies, most have reported linear temperature-fractionation equations with slopes statistically indistinguishable both from each other and from that for inorganic aragonite [2], [77] and have concluded therefore that oxygen isotopes in fish otoliths are deposited at near equilibrium with ambient water. However a common cross-species temperature-otolith δ^18^O fractionation relationship has yet to be defined, the various slopes and intercepts values proposed so far resulting in differences in the estimated temperature of up to 6.1°C (using the equations of [41] and [30]). The difference in slopes and intercept estimates among studies are generally attributed to failure to correctly estimate water temperature or δ^18^O values, or to instrumental artefact (e.g. [42], [43]). However, it has also been suggested that they may reflect physiological ("vital") effects on oxygen uptake, transport and incorporation due to inter-specific differences in physiology and/or adaptation to local environmental conditions [30], [36].

### Potential for vital effects to modify otolith δ^18^O values

Vital effects influencing temperature-δ^18^O fractionation, either kinetic or metabolic, have been implicated in otolith formation by several authors (e.g. [30], [31], [36], [37]). However their impacts on otolith δ^18^O signatures is difficult to evaluate, as the two principle mechanisms covary, through temperature's influence on both fish metabolism [78] and otolith precipitation rate [79], [80]. To date, experimental studies investigating the influence of vital effects on the otolith δ^18^O of juvenile fish [31], [39]–[43], [74] all concluded that otolith δ^18^O was driven mainly by ambient temperatures, and largely independent of fish metabolism [42], [43], [74]. Yet, δ^18^O heterogeneity across temperature-salinity combinations was consistently observed, commonly ranging between 0.5 to 1.5 ‰ for the same rearing conditions (e.g. [39]–[41], [43], [74]), equating to 2.3°C–6.1°C differences in temperature estimates (based on the equation of [2]). This variation may reflect δ^18^O disequilibrium in the otoliths of some, if not all, the fish investigated. If so, then the influence of vital effects on otolith δ^18^O signatures have been largely overlooked to date, as free-ranging fish from genetically distinct stocks will experience more substantial environmental and metabolic variability.

Studies directly investigating the relationship between otolith δ^18^O values and temperature in wild fish have suggested not only inter-specific physiological influences on temperature-otolith δ^18^O fractionation (e.g. [41]–[43]), but also among life-stages and populations of the same species [30], [38], [76]. This conclusion is a logical one, given that body oxygen supply mechanisms and associated tissue, cell and molecular functions are generally temperature adapted, especially in ectotherms like fish [78]. Physiological performance declines on either side of the optimal thermal window, with extreme temperatures resulting in oxygen limitation, hypoxemia and transition to anaerobic mitochondrial metabolism [81]–[83]. This may explain why several studies show non-linear relationships for otolith temperature δ^18^O fractionation [31], [42], [84], [85]. However, in experiments where only a limited portion of the metabolic temperature range is represented and physiological differences between individuals are constrained, the temperature-otolith δ^18^O fractionation may appear to be linear.

Our results bear out this possibility, and further support the idea that species-specific temperature-otolith δ^18^O fractionation relationships may vary with fish physiology linked to their thermal tolerances. Acknowledging the uncertainties resulting from analytical imprecision or inaccurate temporal matching of measured and predicted annual δ^18^O profiles, the fractionation equation of [43] for juvenile plaice produced realistic estimates of otolith δ^18^O for the summer growth of sub-stock A, and the spring and autumn growth of sub-stocks B and C (Figure 5). Oxygen isotope fractionation in our adult free-swimming plaice within the 9–14°C thermal window therefore apparently corresponded with that of juveniles maintained between 11 and 17°C [43]. However, errors in otolith δ^18^O prediction using the equation of [43] across stocks when temperatures were below 9°C and above 14°C (Figure 6), especially the sub-stock specific differences in otolith δ^18^O shifts observed in all of our subject individuals, could not be attributed to any systematic calculation bias (Figure 2 and 7). They suggest non-negligible departure from the juvenile plaice equation beyond the 9–14°C thermal window in adult plaice, with further potential for sub-stock variation in "vital" effects.

Although the occurrence of such "vital" effects requires experimental confirmation, this interpretation is largely consistent with current knowledge of plaice physiology [86] and temperature adaptation in fishes [83], [87]. Indeed, while temperature-dependent physiological principles are unlikely to vary across life stages, functional implications differ between juveniles and adults [78]. Growth optima are expected at lower temperatures in larger fish, reflecting the fact that upper heat limits shift downward [78]. Restricted oxygen supply at extreme temperatures also occurs more readily in adults due to larger body size and reproductive capacity [78]. Likewise, plaice demonstrate a marked decrease in optimum temperature between juvenile and adult stages, from 18–20°C to around 10°C [86]. Because this ontogenetic shift is likely to be accompanied by a downward shift and a reduction of the thermal window allowing oxygen supply [78], it is not surprising that otolith δ^18^O fractionation at 9–14°C in our mature females corresponded to that of juvenile plaice maintained at 11–17°C [43]. Departures from linear otolith δ^18^O fractionation beyond this thermal window are also plausible. Only fish from sub-stocks B and C experienced temperatures above 14°C (in July-September) but, in agreement with expected kinetic "vital" effects on oxygen fractionation [35], [78], otolith δ^18^O values were systematically lower than predicted for these warmer periods. Conversely, temperatures below 9°C were experienced by fish from all three sub-stocks (from December to April in sub-stocks B and C and from December to June in sub-stock A). However, while observed otolith δ^18^O values were lower than predicted in sub-stock C, and probably also sub-stock B, they were surprisingly higher than predicted in sub-stock A. Such sub-stock differences in female plaice metabolism (A *vs*. B and C), at least within the 4–9°C thermal range, are compatible with the metabolic cold adaptation (MCA) hypothesis, which predicts higher metabolic rates of ectotherms from cold environments than those of their more temperate counterparts [88], [89]. Indeed, physiological differences have been demonstrated in same-species populations along a latitudinal temperature gradient [78], [90]. The resulting population-specific patterns of thermal tolerances mean that different metabolic rates would be predicted for different demographic components of the same species when exposed to the same temperature regime [82], [91], [92]. This may partly explain the differences observed between sub-stocks, as sub-stock A has a more clearly delimited northern distribution than B and C (Figure 1).

However, all of the kinetic vital effects described for oxygen isotope fractionation thus far predict that lower metabolic rates should result in higher δ^18^O signatures [35], [79], [80]. In light of the MCA theory, we would therefore expect departure from δ^18^O equilibrium at temperatures below 9°C in plaice to result in higher than expected δ^18^O values for all sub-stocks, with a maximum in the southern sub-stocks (i.e. B and C) due to a more marked decrease in metabolic rate. While measured δ^18^O values at 5°C were indeed higher than predicted for sub-stock A, the opposite was observed for sub-stocks B and C (Figure 6). Failure to adequately sample those growth bands corresponding to the coldest months in the otoliths of the southern sub-stock females may partly be responsible for the observed pattern. Indeed, female North Sea plaice cease feeding for up to five weeks between December and April, due to limited metabolic scope that precludes the simultaneous oxygen demands for spawning and feeding metabolism [49], [57]. Therefore, dilution of the highest δ^18^O signatures (expected January to March, Table 2) by that of warmest months because of reduced otolith growth during spawning might have resulted in the lower than expected otolith δ^18^O values measured in sub-stock B and C. However, differences in physiology in sub-stock A *vs*. B and C during the winter, with potential incorporation of metabolically-derived O^16^ in the otolith of the females of the two southern sub-stocks cannot be fully excluded. If so, the isotopic composition of the otolith organic protein matrix could be involved, since it is mainly comprised of highly oxygenated amino acids [93].

## Conclusions

This study confirmed that, given a reliable isoscape, otolith δ^18^O values can be a highly accurate, low-cost "natural tags" for stock discrimination and broad-scale geolocation of fish, thereby complimenting the results from archival tagging and other population descriptors. However, our results identified patterns that may represent life stage- and population-specific vital effects during oxygen isotope uptake, transport and/or incorporation into otolith aragonite. This casts doubt on the accuracy of past temperature estimates based on otolith δ^18^O from wild fish, even where realistic estimates of water salinity or δ^18^O values were available. We therefore recommend intra-specific validation experiments, involving both different life-stages and (sub-)populations over broad temperature ranges. Indeed, our proposed approach could be transposed to many other species and locations and provide valuable data for conservation and sustainable fisheries management. In many transitory model systems, the relative changes in seawater temperature or salinities are sufficiently pronounced so as to produce markedly distinct otolith δ^18^O signatures and therefore make the accuracy of the estimation of the temperature-δ^18^O relationship irrelevant. Where accuracy is paramount however, the possibility of intra-specific vital effects on oxygen fractionation should be investigated to preclude any bias in the geographical or climatic interpretations of the otolith δ^18^O signals.
