# Correction: Listening In on the Past: What Can Otolith δ^18^O Values Really Tell Us about the Environmental History of Fishes?

**DOI:** 10.1371/journal.pone.0114951

**Published:** 2014-12-01

**Authors:** The PLOS ONE Staff

There is an error in the sixth sentence of the Abstract. The word “of” was inadvertently added. The correct sentence is: However, although largely congruent, measured and predicted annual δ^18^O values did not fully match.

The third and fourth equations in the Material and Methods section are incorrect. Please view the complete, correct equations here: 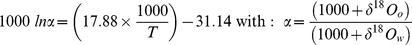
(3)

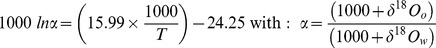
(4)

